# Label-free detection and quantification of ultrafine particulate matter in lung and heart of mouse and evaluation of tissue injury

**DOI:** 10.1186/s12989-022-00493-8

**Published:** 2022-07-26

**Authors:** Saira Hameed, Kun Pan, Wenhua Su, Miles Trupp, Lan Mi, Jinzhuo Zhao

**Affiliations:** 1 Department of Chemistry, Umeå University, 901 87 Umeå, Sweden; 2 Department of Chemistry, Fudan University, Shanghai, 200438 China; 3 Department of Environmental Health, School of Public Health and the Key Laboratory of Public Health Safety, Ministry of Education, Fudan University, 130 Dong’an Road, Box 249, Shanghai, 200032 China; 4 Department of Optical Science and Engineering, Key Laboratory of Micro and Nano Photonic Structures (Ministry of Education), Shanghai Engineering Research Center of Ultra-Precision Optical Manufacturing, Green Photoelectron Platform, Fudan University, 220 Handan Road, Shanghai, 200433 China; 5 Department of Clinical Sciences, Neurosciences, Umeå University, 90185 Umeå, Sweden; 6 IRDR ICoE on Risk Interconnectivity and Governance on Weather/Climate Extremes Impact and Public Health, Fudan University, Shanghai, China

**Keywords:** Fluorescence lifetime imaging microscopy, Scanning electron microscopy, Ultrafine PM, Heart, Lung, Injury

## Abstract

While it is known that air borne ultrafine particulate matter (PM) may pass through the pulmonary circulation of blood at the alveolar level between lung and heart and cross the air-blood barrier, the mechanism and effects are not completely clear. In this study the imaging method fluorescence lifetime imaging microscopy is adopted for visualization with high spatial resolution and quantification of ultrafine PM particles in mouse lung and heart tissues. The results showed that the median numbers of particles in lung of mice exposed to ultrafine particulate matter of diameter less than 2.5 µm was about 2.0 times more than that in the filtered air (FA)-treated mice, and about 1.3 times more in heart of ultrafine PM-treated mice than in FA-treated mice. Interestingly, ultrafine PM particles were more abundant in heart than lung, likely due to how ultrafine PM particles are cleared by phagocytosis and transport via circulation from lungs. Moreover, heart tissues showed inflammation and amyloid deposition. The component analysis of concentrated airborne ultrafine PM particles suggested traffic exhausts and industrial emissions as predominant sources. Our results suggest association of ultrafine PM exposure to chronic lung and heart tissue injuries. The current study supports the contention that industrial air pollution is one of the causative factors for rising levels of chronic pulmonary and cardiac diseases.

**Supplementary Information:**

The online version contains supplementary material available at 10.1186/s12989-022-00493-8.

## Introduction

Air borne ultrafine particulate matter less than 2.5 μm in size (PM particles) is the leading environmental risk factor that impairs metabolic homeostasis [1], and is associated with the morbidity and mortality of cardiopulmonary diseases [2].

Air borne ultrafine PM particles directly enter into the lungs by inhalation and are transported into extrapulmonary organs such as kidney, and liver, [3], including brain [4]. The quantification of ultrafine PM particles can contribute to elucidation of the effects of the PM particles on different tissues and organs. Due to nonspecific and uncommon respiratory symptoms, clinical discoveries depend on evaluation of the involvement of additional organs [5]. In recent studies magnetic iron oxide (Fe_3_O_4_) known as magnetite has been detected in neurodegenerative tissues with correlation between the amount of brain magnetite and the incidence of Alzheimer’s disease [6, 7]. Our previous study has shown that ambient PM particles reach mouse brain due to permeability of the blood brain barrier, resulting in neuroinflammation, tangles and plaque formation similar to Alzheimer’s disease [4]. Carbon black particles have been detected in human placental tissue crossing the blood placental barrier [8], and magnetite has been detected in human brain [9], indicating that ultrafine PM particles can enter into different organ systems [10].

However, it is difficult to detect the amount of PM particles in different tissues because of the variable size and complex chemical nature. Consequently, there are few studies focused on detecting the amount of ultrafine PM particles in different tissues to determine the rates of deposition. It is generally accepted that there is a large amount of black carbon in the air borne particulate matter. There are several reports of carbon particles with high two-photon absorption cross-section, ranging between 39,000 and 48,000 GM (Goeppert-Mayer unit, with 1 GM = 10^–50^ cm^4^ s/photon) [11–13]. In contrast, the endogenous fluorescent probe, NADH was reported to have a much lower two-photon absorption cross-section, around 340 GM [14]. Thus, black carbon can be an efficient two-photon fluorescent indicator of ultrafine PM particles with the femtosecond pulsed laser excitation. In 2019, Bové et al. performed fluorescence spectroscopy, fluorescence lifetime analysis, and two-photon excitation imaging on human placental tissues [8], which are similar to methods we used in this paper. They confirmed the carbonaceous nature of the identified black carbon particles and external contamination of the tissues could be excluded. And they found a positive association between the placental black carbon load and the mothers' residential black carbon exposure. Therefore, the two-photon excitation fluorescence lifetime imaging method is reliable for measuring the carbon particles in tissues.

In this study, sixteen mice were divided into two groups and were exposed to concentrated ultrafine PM particles (PM, dirty air), and filtered air (FA, control), using the “Shanghai Meteorological and Environmental Animal Exposure System (Shanghai-METAS)”, located in the School of Public Health at Fudan University at Xujiahui District in Shanghai. We used field emission scanning electron microscopy (FE-SEM) to visualize ultrafine PM particles, and fluorescence lifetime imaging microscopy (FLIM) to quantify the PM particles in lung and heart tissues.

Knowing the precise deposition process and the transport mechanism of inhalable particles is crucial for health risk assessment and evaluation of target organ injury [15]. The deposition of ultrafine PM particles in different tissues has adverse effects on the target organs including tissue injury and inflammation [16] that are causally linked to cardiopulmonary diseases such as atherosclerosis, coronary heart disease and chronic obstructive pulmonary disease. Under the influence of pathological states healthy proteins lose their normal structure and function and aggregate in tissue and organs in the form of amyloid deposits. Amyloids can accumulate not only in brain but also in different tissues and body organs that result in clinical syndromes [17]. Studies have shown that exposure to ultrafine PM particles led to influx of inflammatory cytokines in serum, heart, liver and lung of mice [18–21]. Our recent study has shown that exposure to ambient PM particles resulted in inflammation, deposition of Aβ amyloids and formation of neurofibrillary tangles and plaques in mouse brain [4]. Amyloids can accumulate not only in brain but also in different tissues and body organs that result in clinical syndromes [17]. A recent study has shown multiorgan amyloidosis in a coal miner [22]. In a Swedish study on amyloidosis, all of the 33 cases had simultaneous pulmonary and cardiac involvements [5]. A previous report has suggested that amyloid protein could be produced in tissues and might be derived from precursors in the blood circulation [23].

## Results

### Detection and quantification of ultrafine PM particles in lung and heart tissues by fluorescence lifetime imaging microscopy (FLIM)

**Fig. 1:**
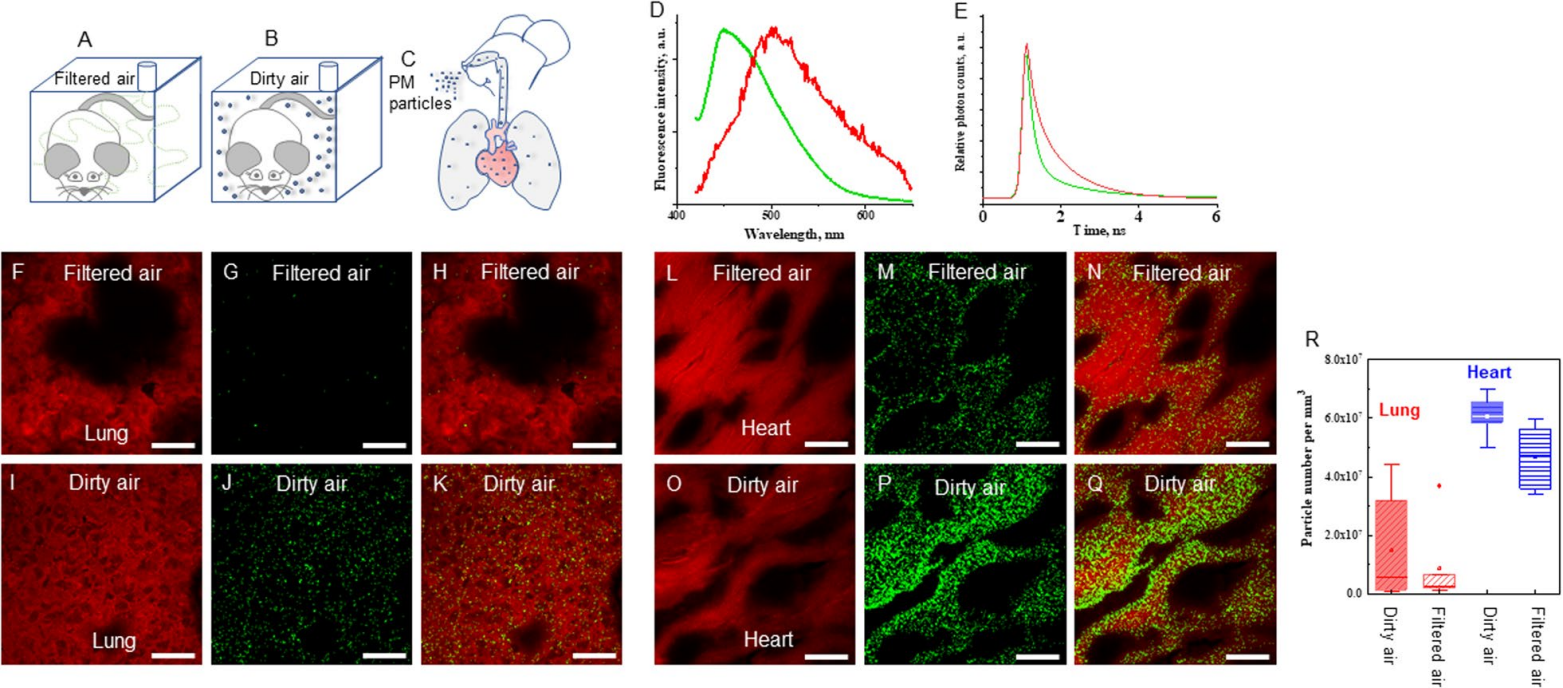
Mice were kept in exposure chambers for six months. Graphical illustrations **A**) Mouse in filtered air chamber. **B**) Mouse in dirty air chamber. **C**) Movement of ultrafine PM particles into the respiratory track and accumulation in lungs and heart. **D**) Normalized fluorescence spectra of ultrafine PM in PBS (green) and auto-fluorescence of tissues (red). **E**) Typical lifetime decay curves of ultrafine PM in tissues (green) and auto-fluorescence of tissues (red). **F**) Auto-fluorescent image of lung tissue from filtered air. **G**) Ultrafine PM particles from filtered air. **H**) Fluorescent deposition pattern of ultrafine PM particles in lung tissue from filtered air. **I**) Auto-fluorescent image of lung tissue from dirty air. **J**) Ultrafine PM particles from dirty air. **K**) Fluorescent deposition pattern of ultrafine PM particles in lung tissue from dirty air. **L**) Auto-fluorescent image of heart tissue from filtered air. **M**) Green dots are the ultrafine PM particles. **N**) Fluorescent deposition pattern of ultrafine PM particles in heart tissue from filtered air. **O**) Auto-fluorescent image of heart tissue from dirty air. **P**) Green dots are the ultrafine PM particles. **Q**) Fluorescent deposition pattern of ultrafine PM particles in heart tissue from dirty air. Scale bar: 20 µm. The resolution of FLIM images is approximately 250 nm. **R**) The estimated particle density in lung and heart of mice

Sixteen mice were divided equally into filtered air (FA) (control) (Fig. 1A), and 2X concentrated air (Fig. 1B) groups, and given the FA or concentrated ultrafine PM exposure (dirty air). The mean concentrations of ultrafine PM in dirty air and FA chambers during the exposure were 71.20 ± 45.01 and 11.76 ± 4.40 μg/m^3^, respectively. The mean outdoor ultrafine PM concentration during the exposure was 43.00 ± 6.05 μg/m^3^. We hypothesize that during respiration ultrafine PM particles from air pollution entered into lungs and were carried to heart via blood circulation. Figure 1C presents a graphical illustration showing the movement of ultrafine PM particles inside the respiratory and blood circulation systems. FLIM microscopy enabled label-free detection and quantification of ultrafine PM particles on lung and heart tissues of mouse. Fluorescence spectra of ultrafine PM particles in PBS and tissues showed large overlap between 450 and 550 nm (Fig. 1D). Thus, it is hard to distinguish ultrafine PM from the tissues. However, the fluorescence lifetime of ultrafine PM and tissues were quite different, the lifetime of ultrafine PM was much shorter than that of tissues (Fig. 1E). Based on the different lifetime values, the green dots denote PM particles in tissues and the red fluorescence reveals the tissue structure, and the superimposed images showed clear distribution of PM particles (Fig. 1F–Q). During respiration, the ultrafine PM particles from dirty air enter directly into lungs and alveoli lined with blood capillaries. Lung tissues from the filtered air group showed little deposition of ultrafine PM particles (Fig. 1H). Lung tissues from the dirty air exposure group showed large numbers of ultrafine PM particles (Fig. 1K). The blood capillaries absorb oxygen and ultrafine PM particles from lung during respiration and transport to heart (Fig. 1N). Heart absorbs and collects higher amounts of ultrafine PM particles from dirty air (Fig. 1Q). The particle densities in lung and heart of mice were estimated (Fig. 1R) accordingly. The results showed that the median numbers of particles in lung of ultrafine PM-treated mice (dirty air group) are 3.4 times more than that in the FA-treated mice, and in heart of ultrafine PM-treated mice, 1.3 times more than in FA-treated mice. The dispersion of data in lung tissues is relatively large across the animals.

### Exposure to concentrated ultrafine PM particles caused lung damage

**Fig. 2:**
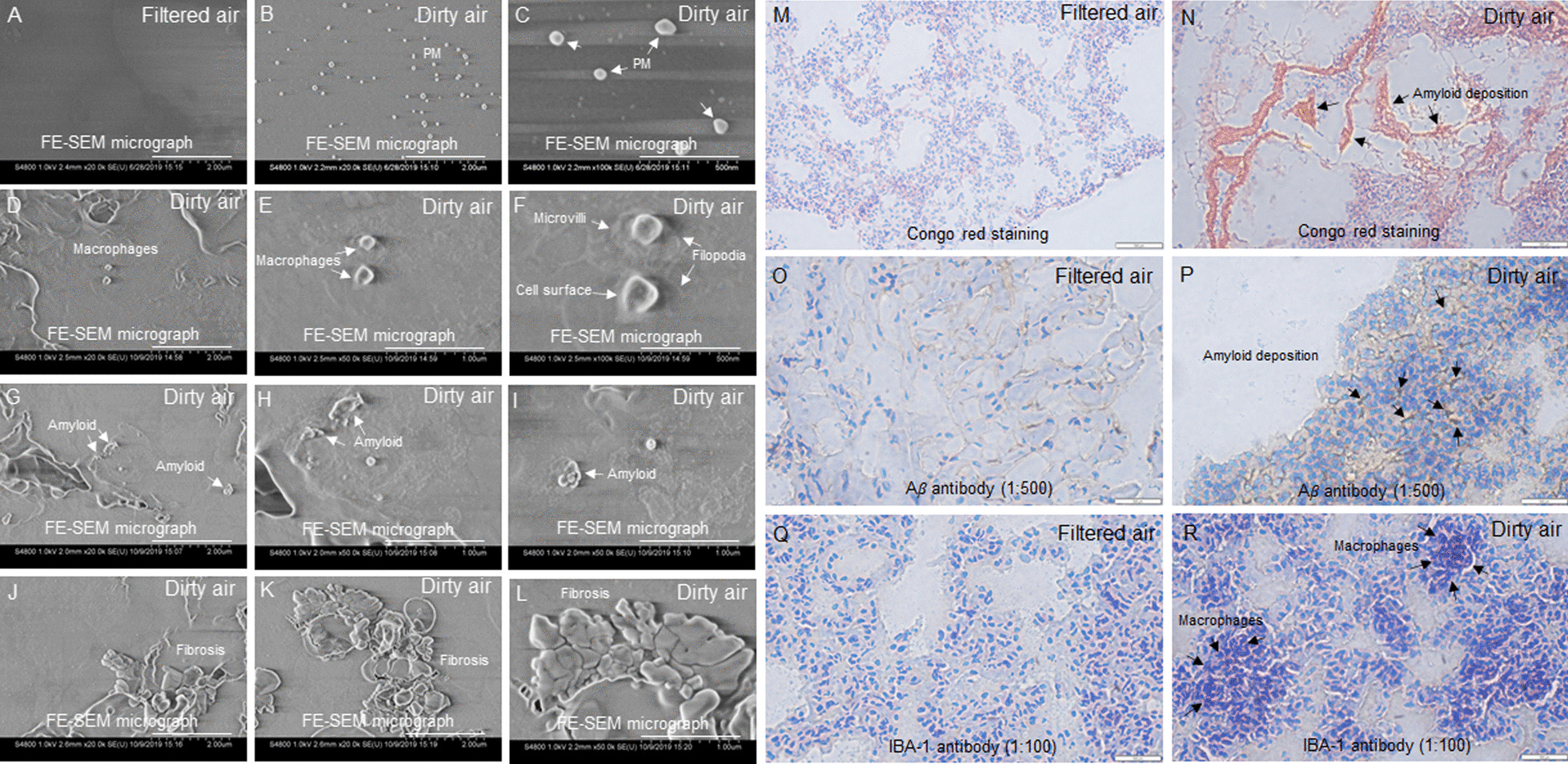
Field emission scanning electron microscopy (FE-SEM) of lung tissues. **A**) Lung tissues from filtered air showed no signs of abnormality, whereas **B**, **C** lung tissue sections from dirty air exposure group showed ultrafine PM particles, **D**–**F** macrophages. High magnification FE-SEM image of the macrophages showed cell surface, knob like microvilli, and filopodia that extended outwards from periphery of the cells (**F**). Lung tissue sections from dirty air exposure group showed amyloid deposits (**G**–**I**), and fibrosis (**J**–**L**) Congo red staining, **M** lung tissues from filtered air (control) showed no signs of abnormality, whereas **N** lung tissue from the dirty air showed amyloid deposition. Immunohistochemistry with amyloid marker Aβ antibody (1:500), **O** from lung tissue sections from filtered air (control) group showed no signs of abnormality, whereas **P** lung tissue from dirty air showed immunoreactive areas with dark brown amyloid deposits. Immunohistochemistry with macrophage marker IBA-1 antibody (1:100), **Q** lung tissues from filtered air showed no signs of abnormality, whereas **R** lung tissue from dirty air showed macrophages. Magnification (**A, B, D, G, J, and K**) 20 k, scale bar: 2 µm. Magnification (**E, H, I,** and **L**) 50 k, Scale bar: 1 µm. Magnification (**C** and **F**) 100 k, Scale bar: 500 nm. Magnification (**M** and **N**) 20X, Scale bar: 500 µm. Magnification (**O**, **P**, **Q**, and **R**) 40X, Scale bar 500 µm

Surface evaluation of tissues by field emission scanning electron microscopy (FE-SEM) showed that lung tissue sections from filtered air (control) group showed no signs of abnormality (Fig. 2A), whereas ultrafine PM particles (Fig. 2B–C), macrophages (Fig. 2D–F), and amyloid deposits (Fig. 2G–I), and fibrosis (Fig. 2J–L) were detected in lung tissue sections from the dirty air exposure group. The effects of ultrafine PM particles on lung tissue sections were determined by histopathological evaluations. Congo red (azo dye) was used as a classical qualitative method to stain and detect amyloid buildup in tissue sections. Congo red staining of lung tissues from filtered air showed no signs of abnormality (Fig. 2M), whereas lung tissue from dirty air showed salmon red amyloid deposits under light microscopy (Fig. 2N). Moreover, immunohistochemistry with Aβ antibody was used to enhance sensitivity of amyloid detection that found no signs of abnormality in lung tissues from filtered air (control) group (Fig. 2O), whereas lung tissue from dirty air showed immunoreactive areas with dark brown amyloid deposits (Fig. 2P). The AIF-1/IBA-1 antibody was used as a macrophage marker in tissue injury. AIF-1 is cytosolic actin binding protein allograft inflammatory factor also known as IBA-1 or calcium binding adapter molecule 1 s. Immunohistochemistry with IBA-1 antibody found no signs of abnormality in lung tissues from filtered air (Fig. 2Q), whereas macrophages were detected in lung tissues from dirty air exposure group (Fig. 2R).

### Exposure to concentrated ultrafine PM particles caused heart tissue damage

**Fig. 3:**
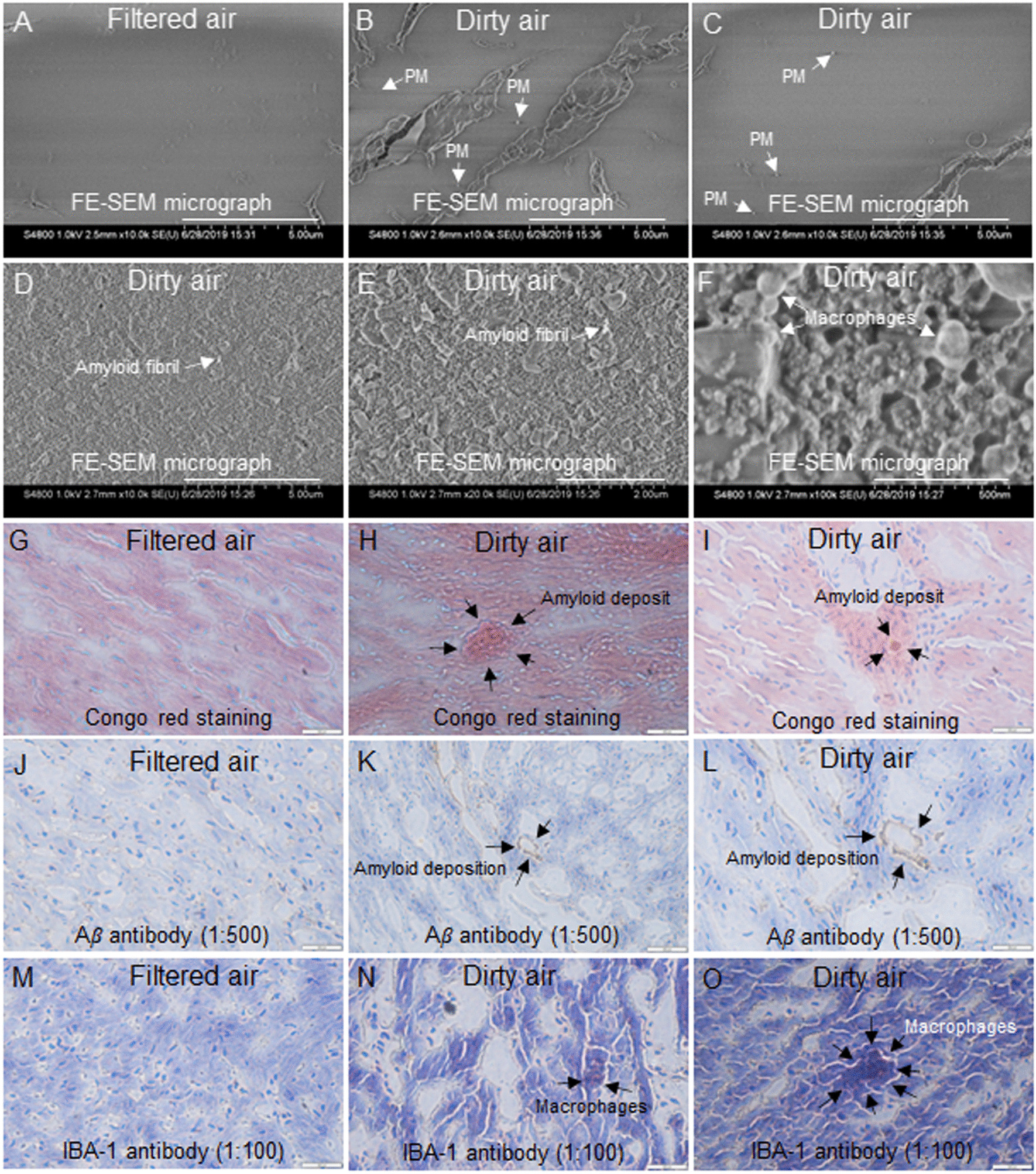
Field emission scanning electron microscopy (FE-SEM) of heart tissues. **A** Heart tissues from filtered air (control) group showed no signs of abnormality, whereas **B**, **C** heart tissue from dirty air exposure group showed ultrafine PM particles, **D**, **E** amyloid, and **F** macrophages. Histopathological evaluation by Congo red staining, **G** heart tissues from filtered air (control) group showed no signs of abnormality, whereas **H**, **I** heart tissue from the dirty air showed amyloid deposition. Immunohistochemistry with amyloid marker Aβ antibody (1:500), **J** from heart of filtered air (control) group showed no signs of abnormality, whereas) heart tissue from dirty air showed immunoreactive areas with dark brown amyloid deposition (**K** and **L**). Immunohistochemistry with macrophage marker IBA-1 antibody (1:100), **M** heart tissues from filtered air (control) group showed no signs of abnormality, whereas (**N** and **O**) heart tissue from dirty air showed macrophages. Magnification (**A** to **D**) 10 k, scale bar: 5 μm. Magnification (**E**) 20 k, scale bar 2 μm. Magnification (**F**) 100 k, scale bar: 500 nm. Magnification (**G, H, I, J, L, M, N,** and **O**) 40X, Scale bar: 500 µm. Magnification (**K**) 20X, Scale bar: 500 µm

Surface evaluation of tissues by FE-SEM showed that heart tissue sections from the filtered air (control) group showed no signs of abnormality (Fig. 3A), whereas ultrafine PM particles (Fig. 3B, C), amyloid (Fig. 3D, E), and macrophages (Fig. 3F) were detected in heart tissue sections from dirty air exposure group. Moreover, the effects of ultrafine PM particles on heart tissue sections were determined by histopathological evaluations. Congo red staining of heart tissues from filtered air (control) group showed no signs of abnormality (Fig. 3G), whereas heart tissue sections from dirty air showed amyloid deposition (Fig. 3H, I). Moreover, immunohistochemistry with amyloid marker Aβ antibody found no signs of abnormality in heart tissues from filtered air (control) group (Fig. 3J), whereas heart tissue from dirty air showed amyloid deposition (Fig. 3K, L). Immunohistochemistry with macrophage marker IBA-1 antibody found no signs of abnormality in heart tissues from filtered air (control) group (Fig. 3M), whereas macrophages were detected in heart tissues from the dirty air exposure group (Fig. 3N, O).

### The organic components of ultrafine PM particles in dirty air and FA chambers

The organic molecular formulae in dirty air and FA were detected by electrospray ionization mass spectrometry (ESI–MS). Electro-spray ionization (ESI) is a powerful technique for analysis of molecules at different polarities in a complex sample mixture [24]. In positive ion mode (ESI +) the spraying nozzle is kept at positive potential and protonation of the analyte occurs. While in the negative ion mode (ESI-) the spraying nozzle is kept at negative potential and deprotonation of the analyte occurs.

**Fig. 4:**
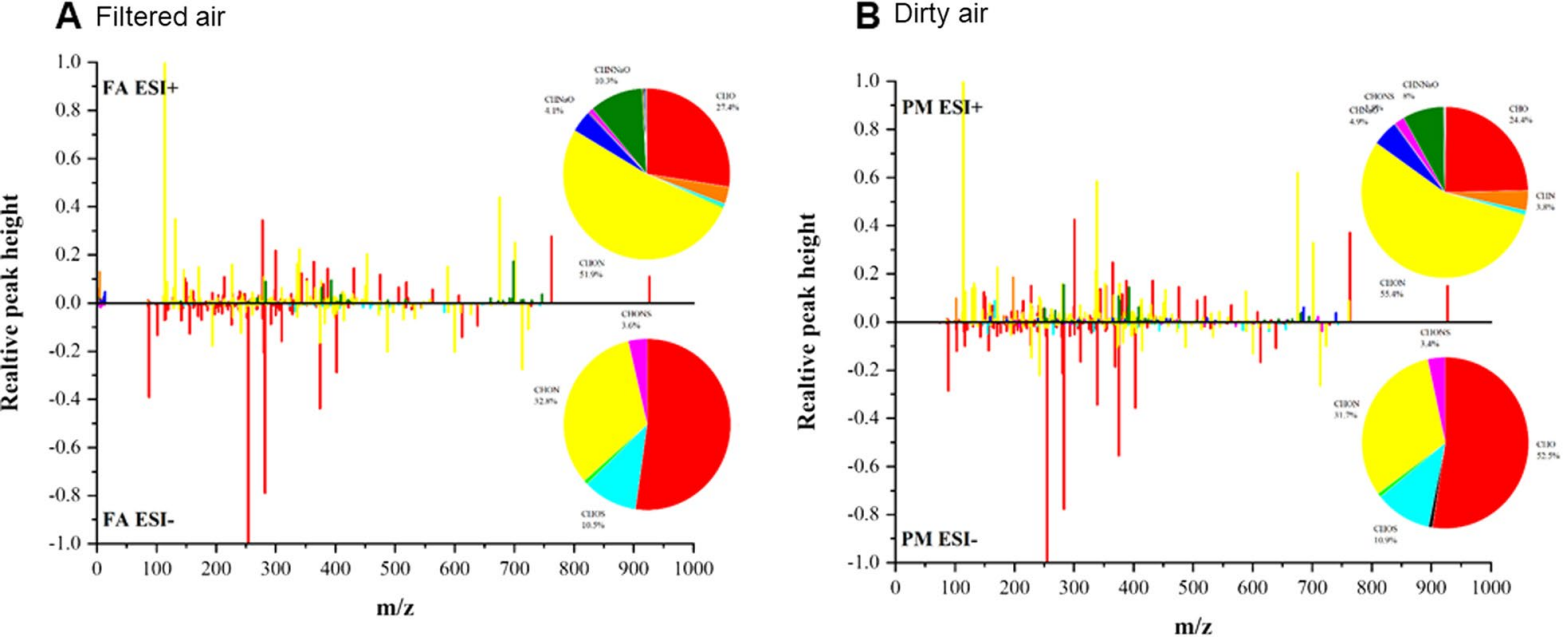
Mass spectrometry anslyses of ultrafine PM particles

The Fig. 4A, B show the mass spectra of samples and the ratios of different element compositions in FA and dirty air groups. The numbers of organic molecular formulae in dirty air and FA are shown in Tables 1 and 2. The results indicated that dirty air group showed more organic substances, characterized by CHON, CHNaO, CHNNa, CHONS, CHNNaO, CHO, CHOS and CHONS when compared with FA group.

**Table 1:** The number of molecular formulae in FA and dirty air at positive ion mode (ESI +)

Element Combination	FA (Mean ± SD)	Dirty air (PM) (Mean ± SD)
CHO	83.1 ± 4.6	103.8 ± 5.3
CHN	11.3 ± 1.1	16.4 ± 2.4
CHOS	1.8 ± 0.6	3.2 ± 0.8
CHNS	1.1 ± 0.4	1.2 ± 0.1
CHON	135.7 ± 11.7	236.4 ± 13.5
CHNaO	14.2 ± 2.1	20.6 ± 3.3
CHNNa	0.5 ± 0.2	0.8 ± 0.5
CHONS	3.2 ± 0.8	8.3 ± 1.2
CHNNaO	35.2 ± 3.6	33.7 ± 4.7
CHONaS	1.2 ± 0.5	0 ± 0
CHNNaS	1.3 ± 0.6	0 ± 0
CHONSNa	0.8 ± 0.2	1.3 ± 0.3
Total	288.7 ± 17.3	426.4 ± 20.7

**Table 2:** The number of molecular formulae in FA and dirty air at negative ion mode (ESI-)

Element Combination	FA (Mean ± SD)	Dirty air (PM) (Mean ± SD)
CHO	89.1 ± 6.3	138.8 ± 8.4
CHS	0 ± 0	2.1 ± 0.7
CHN	0 ± 0	0 ± 0
CHOS	25.6 ± 3.3	28.9 ± 3.6
CHNS	2.2 ± 0.7	2.3 ± 0.4
CHON	71.2 ± 9.2	83.5 ± 6.9
CHONS	9.4 ± 1.5	9.3 ± 2.1
Total	196.8 ± 10.8	265.1 ± 13.5

Tables 1 and 2 The number of organic molecular formulae in air samples at positive and negative modes (ESI + and ESI-) in (**A**) filtered air (FA), and (**B**) dirty air. An Agilent 1200 series HPLC with a C18 column (SB-C18, 3.0 × 100 mm, 1.8 μm) was used for chromatographic separation. At ESI + , a: C_6_H_11_NO; b: C_6_H_13_NO_2_; c: C_16_H_22_O_4_; d: C_22_H_43_NO; e: C_22_H_42_O_4_; At ESI-, A: C_3_H_6_O_3_; B: C_16_H_32_O_2_; C: C_18_H_36_O_2_; D: C_20_H_40_O_6_; E: C_22_H_44_O_6_; F: C_24_H_44_N_4_O_4_; G: C_30_H_55_N_5_O_5_; H: C_35_H_70_N_2_O_10_.

### The metal components of ultrafine PM particles in dirty air and FA chambers

The samples of ultrafine PM particles in dirty air and FA chambers were collected weekly during the exposure. Because of the hazards of heavy metals in air borne particulate matter, we measured 8 heavy metals including Zn, Bi, Cd, Ni, Fe, Mn, Cr and Cu by ICP-MS in this study. Additional file 1: Fig. S1 shows the fractograms of the 8 metals. As shown in Additional file 2: Table S1, the concentrations of these 8 elemental components in dirty air were about five times more than those in the FA chamber, demonstrating that the METAS we used to give the mice dirty air and FA exposure significantly concentrated the ambient ultrafine PM particles without changing its components. Of these 8 metals, Fe, Zn, Cd and Mn were the main components.

## Discussion

Numerous studies have indicated the association between air borne particulate matter and diseases in humans and experimental animals. This includes cardiopulmonary diseases, diabetes, cerebrovascular diseases and reproductive toxicity [25, 26]. Inhalation of ultrafine PM particles have adverse health effects. Exposure to PM_2.5_ over shorter periods of time reduced lung function in children [27].

As the main air pollutant, the association between ultrafine particulate matter (PM particles) and health is of great concern. This is particularly problematic in heavily industrialized and populated areas, with China often identified as a region with the highest ultrafine PM concentration in the world [28]. In 2020, the annual average concentration of ultrafine PM in China was 36 μg/m^3^, lower than that in 2017 (43 μg/m^3^), but still higher than both Chinese air quality standard (35 μg/m^3^) and U.S EPA standard (12 μg/m^3^) [28].

In this study, the mice were raised in groups and were allowed to eat food and drink water freely inside whole body exposure chambers METAS [29], for filtered air (control) and dirty air, between 2018 and 2019. The concentrations in dirty air and FA chambers were 71.20 μg/m^3^ and 11.76 μg/m^3^, respectively. The mice were sacrificed after six months of exposure in 2019, and their organs were extracted for analyses. Our recent study focused on brain of those mice [4]. In the current study, lung and heart tissues from the same group of mice were used for analyses.

Airborne ultrafine PM particles enter into lung tissue by passive transport along surface liquids, and phagocytosis within alveolar macrophages [30]. Although several lines of evidence support the theory that particles translocate from lung into the circulation, then enter into heart, liver and kidney, quantifying the particles in these organs took precedence in assessing particle toxicological effects. Previous studies have used second harmonic generation (SHG) to detect black carbon in placenta [8] and high-resolution transmission electron microscopy (HRTEM) to detect magnetite in brain [9], but there are few studies to quantify the amounts of ultrafine PM particles in tissues. It is noteworthy that airborne ultrafine PM particles was a mixture and not a single chemical, which made the detection more difficult.

In this study, FLIM enabled label free detection and quantification of ultrafine PM particles with high resolution on lung and heart tissues, which can provide broad insights into the distribution of particles entering into tissues. Although there is the limitation that FLIM can’t completely separate the debris of cellular and tissue injury/inflammation from ultrafine particles, compared with transmission electron microscopy (TEM), FLIM has several advantages: sample preparation is simple and does not need special treatment; the imaging range of FLIM is larger than TEM, which is convenient for large samples statistics. In this study ultrafine PM particles were also detected in the filtered air group because filters do not have 100 percent efficiency, and extremely small particles can pass through them. Our current study found that the amounts of ultrafine PM particles was higher in dirty air (PM) group than that in FA group. Intriguingly, ultrafine PM particles were more in heart than lung, probably because lung has air sacs and the PM particles are cleared by transportation over fluid and phagocytosis [30], whereas heart is the muscular organ that pumps oxygenated blood along with dissolved PM particles from lungs. To the best of our knowledge it is the first report on label-free detection and quantification of ultrafine PM particles in lung and heart tissues of mouse, and the mechanism of particle distribution is unknown. Previous studies have shown that PM particles enter in blood circulation just after exposure [31]. As heart is the main pumping organ therefore it is hypothesized that the powerful mechanical force of blood passing through heart may contribute to adsorption of ultrafine PM particles into heart muscles, which may act as a sink for inhaled particulate matter. However, it should be taken into consideration that as there is no known evidence from literature to support the hypothesis therefore future studies are needed to give a more definitive statement how ultrafine PM particles enter into heart tissue. As the number of ultrafine particles were higher in heart as compared to lung, therefore, it may have more adverse effects on heart as compared to lung of mouse. Interestingly, a recent report on certain cardiopulmonary ailments with pathological evaluations of lung and heart tissues of 76 patients by Mayo clinic physicians have shown that cardiac manifestations might occur earlier and are more frequent and severe than pulmonary disease, and the survival rate of patients was found to be directly related to the degree of cardiac involvements [5].

Scanning electron microscopy provides a unique means for examining the dynamic aspects of inflammatory response. FE-SEM enabled detection of ultrafine PM particles (Fig. 2B, C), macrophages (Fig. 2D–F), amyloid deposition (Fig. 2G–I), and fibrosis (Fig. 2J–L) on lung tissues from dirty air exposure group. Under the influence of pathological states healthy proteins lose their normal structure and function and aggregate in tissue and organs in the form of amyloid deposits that can be detected by the classical gold standard congo red (azo dye) staining that gives salmon red color under light microscopy [17]. Moreover, sensitivity of detection was enhanced by immunohistochemical staining with amyloid marker Aβ antibody that detected amyloid deposition (Fig. 2N, P), and macrophage marker IBA-1 antibody that detected macrophages at sites of tissue injury (Fig. 2R). Previous studies have shown that ultrafine PM exposure was associated with the release of inflammatory cytokines and inflammatory cell infiltration. A recent study has shown that activation of the NLRP3/ caspase-1 signaling pathway by ultrafine PM particles induced pulmonary inflammation [32]. The mechanism of inflammation in pulmonary diseases has been reviewed recently [33]. Inflammatory response has been certified as a vital mechanism linking particulate matter and adverse effects, and our results directly verified the occurrence of inflammation. Chronic inflammation and amyloidosis directly target lungs, severely effect alveolar structures, hamper gaseous exchange and result in serious respiratory impairment including asthma and other pulmonary diseases [33]. Pulmonary inflammation is a risk factor for cardiovascular diseases [34]. In this study surface evaluations by FE-SEM detected ultrafine PM particles (Fig. 3B, C), amyloid (Fig. 3D, E), and macrophages (Fig. 3F) in heart tissues from dirty air exposure group. The observations were supported by histochemical evaluations that also detected amyloid deposits (Fig. 3H, L) in the lung tissues. Heart is composed of heterogenous population of cells [35]. Macrophages are found at the site of fibrosis, that activate reparative and maladaptive processes that lead to organ dysfunction in many different diseases [36, 37].

Inflammation is common in coronary heart diseases and atherosclerosis but the mechanism is not known. Moreover, amyloids can infiltrate heart tissues and result in cardiac amyloidosis. Cardiac amyloidosis results in myocardial thickening and dysfunction [38]. The results from the current study suggests that air pollution is one of the causative factors for rising levels of chronic pulmonary and cardiac diseases [39, 40] and that particulate induced amyloidosis is a potential mechanism for targeted therapeutic development.

A previous human study found metal components (e.g. Al, Fe, Ca, Ni, Cu, Pb, V and Zn) of PM_2.5_ significantly decreased whole blood coagulation time in healthy subjects [41]. PM_2.5_ rich in metal components such as nickel (Ni) has been linked to adverse cardiopulmonary effects [42, 43]. Moreover, other studies also found that metal constituents such as Zn, Cd, Mn, Cu [44], Cd, Pb [45] in PM_2.5_ were associated with a variety of adverse health effects. In this study, we determined eight main (Zn, Bi, Cd, Ni, Fe, Mn, Cr and Cu) metals in ultrafine PM particles, in which the concentration of Fe, Zn, Cd and Mn are higher than other metals. The variations in molecular compositions of ultrafine PM particles in dirty air and FA were also evaluated in this study. Sulphur and nitrogen containing organics have received the most attention because they can be used to reveal the pollutant sources, and aging mechanisms. CHON species that can form via gas-phase nitrate radical initiated oxidation are also significant components of secondary organic aerosol [46]. A previous study indicated that CHN species were a significant contributor to the organic matter at the Beijing site, and high levels of CHN species and their CH2 homologous series were identified as quinoline and benzo [f] quinoline compounds, which may have considerable health implications [47]. Therefore, chemical characteristics of organic aerosols can provide a clue for exploring the adverse effects of ambient particulate matter. Moreover, the components of air pollution are associated to increased hospital visits for pulmonary and cardiac ailments [48].

We want to emphasize that ultrafine PM particles are complex mixture of chemicals of variable sizes that range from course to ultrafine [49]. The nature of PM particles may change as they enter inside the living organisms. PM particles cause the activation of oxidative stress and generation of reactive oxygen species [49]. Moreover, it is very important to consider soluble components of PM particles during interpretation of the results of this study. The ultrafine PM particles detected in lung and heart tissues could be the insoluble fraction of ultrafine particles that once inside the body may be coated by biomolecules such and form protein corona and soluble ions of metal complexes. We also tried scanning electron microscopy / energy dispersive X-Ray spectroscopy (SEM/EDS) to perform elemental/ component analysis of ultrafine PM particles in lung and heart tissue sections, but it was not successful because the PM particles were scattered over tissue sections as tiny particles in nm size range, and no big clusters were found.

Moreover, as mice were exposed to ultrafine PM particles with concentrations about two times higher than ambient air in the Shanghai metropolitan areas, and the control group was exposed to air that passed through HEPA filters to remove most of the ultrafine particles. While, the gaseous contents in both chambers were same. Therefore, it is suggested that the tissue damages in the lung and heart tissues observed in dirty air exposure group were seeded by ultrafine PM particles, which were lower in filtered air (FA) control group.

## Conclusion

In this study fluorescence lifetime imaging microscopy (FLIM) enabled label-free detection and quantification of ultrafine PM particles on lung and heart tissues of mouse. Field emission scanning electron microscopy (FE-SEM) presented visuals of ultrafine particles on tissues, and histological insights on toxicological effects of ultrafine PM exposure to chronic lung and heart injuries were presented. It suggested that rising levels of air pollution are among the causative factors associated with increased cardiopulmonary disorders worldwide.

## Materials and methods

### Animal management

Six weeks old *Mus musculus* (C57BL/6 male mice) were purchased from Shanghai Jiesijie Laboratory Animal Co., Ltd (Shanghai, China). They were housed in a pathogen-free animal facility at Fudan University, at constant temperature (21 °C ± 1 °C) and humidity (60%) on a day and night cycle of 12 h each, and were maintained on normal chow diet. The procedures were approved by the Institutional Research Committees of the Fudan University, Shanghai, China, and the methods were performed in accordance with the set regulations and guidelines.

### Exposure of concentrated ultrafine PM particles to mice

Sixteen mice were divided into two groups and were exposed to concentrated ultrafine PM (PM, dirty air), and filtered air (FA, control), in whole body exposure chambers, using the “Shanghai Meteorological and Environmental Animal Exposure System (Shanghai-METAS)”, located in the School of Public Health at Fudan University at Xujiahui District in Shanghai. Ambient air passed through HEPA filters to get filtered air [50]. In the dirty air exposure chambers Shanghai-METAS, only the particles with diameters less than 2.5 µm entered into the chamber. The versatile aerosol concentration enrichment system (VACES) was used for enrichment of ultrafine PM particles in the dirty air exposure chamber. We have used the exposure system to perform several studies [29, 51].

In this study, the exposure lasted for 8 h per day, 6 days per week, in a total of 24 weeks. The mice were freely allowed to eat food and drink water in whole body exposure chambers.

### The real-time concentration of ultrafine PM particles

The real-time concentrations of ultrafine PM particles from exposure chamber and control chamber were continuously measured by TEOM (Thermo Fisher Scientific, Waltham, MA), and ultrafine PM particles were sampled on Teflon filters (Gelman Teflon, 37 mm, 0.2 mm pore) for subsequent measuring the accurate concentrations and the components such as constituents of polycyclic aromatic hydrocarbons (PAHs) and trace metals.

### Metal concentration and component analysis of ultrafine PM particles

The Teflon filter (Gelman Teflon, 37 mm, 0.2 mm pore) with ultrafine PM particles was cut and divided into two parts. The filters were treated with 10 mL of 60% high-purity nitric acid (HNO_3_) and 3 mL of 37% perchloric acid (HClO_4_). The solutions containing filters were heated in microwave for 1 h. They were then stored at 4 °C until analysis. The metal concentrations of ultrafine PM particles were determined by inductively coupled plasma mass spectrometry (ICP-MS).

### Organic combination analysis of ultrafine PM particles in dirty air and FA

The filters with ultrafine PM particles were cut into pieces using scissors and extracted in 30 mL methanol under ultrasonication for 30 min. The extracted solution was filtered (polytetrafluoroethylene membrane) through a syringe with 0.22 μm pore size. After concentration, the final volume was 1 ml prior to HPLC–DAD-Q-TOF–MS analysis. An Agilent 1200 series HPLC with a C18 column (SB-C18, 3.0 × 100 mm, 1.8 μm) was used for chromatographic separation with an injection volume of 2 μL. The flow rate was set to 0.4 mL/min and the gradient separation was conducted with 0.1% formic acid in water (A) and methanol (B). The concentration of B was 5% for the first 0.5 min increased to 95% from 0.5 to 27 min, and then decreased back to 5% from 27 to 27.1 min. The identification of BrC was determined with an Agilent 6520 Q-TOF–MS and an Agilent G1315D diode array detector (DAD). UV–Vis absorption was measured using the DAD detector over the wavelength range of 190–600 nm. The TOF–MS was equipped with electrospray ionization (ESI), operated in both positive and negative ion modes. The drying gas flow rate was 7 L/min, and the temperature and flow rate of sheath gas were 350 °C and 11 L/min, respectively.

This study presents the molecular composition of ultrafine PM particles according to the protocol presented in Daellenbach et al. [52]. For the whole component analyses, the allowed range or the atomic number limit of carbon, hydrogen, oxygen, nitrogen and sulphur in the molecular formulae were 1–100, 1–200, 0–50, 0–5 and 0–2, respectively. The molecular compositions were assigned to the signals using a tolerance level ± 2 ppm. The generated formulas satisfied elemental rules: O:C ≤ 1.5; 0.3 ≤ H/C ≤ 2.5; 0 ≤ N/C ≤ 0.5; 0 ≤ S/C ≤ 0.2., and irregular formulae were excluded.

### Preparation of lung and heart tissue sections

The protocol approved by the institutional review board was followed, the mice were sacrificed, and lung and heart samples were collected and immediately stored at -80 °C until sectioning. The samples were attached to the aluminum disc and 10-µm-thick tissue slices were prepared using Leica CM1950 cryostat (Leica Biosystems), attached over the surface of adhesion microscopic glass slides, and stored in airtight falcon tubes at -80 °C before analyses.

### Field emission scanning electron microscopy (FE-SEM)

The falcon tubes containing glass slides of heart or lung cryo-sections were dried at room temperature before analysis without any pretreatment. The surface of the FE-SEM aluminum sample stage was covered with carbon conducting tape and the glass slide was attached onto it. A Hitachi S-4800 field emission scanning electron microscope (FE-SEM) equipped with Bruker Xflash 6160 detector was used for observation of heart or lung tissue sections of eight mice from dirty air and eight mice from filtered air, at acceleration voltage of 1.0 kV, and emission current of 10 µA. The vacuum level in the observation chamber was ~ 10^−7^ Pa. The observations were made at the working distance of 2.1 mm to 2.4 mm, and at the scan speed of 20 s for each figure, at 10 k, 20 k, 50 k, and 100 k magnifications.

### Fluorescence lifetime imaging microscopy (FLIM)

The fluorescence spectra of ultrafine PM particles in PBS solution and the tissues were excited by a 405 nm CW laser (BDL-405-SMC, Becker and Hickl, Berlin, Germany) and recorded by an optical fiber spectrometer (Ocean Optics, USB2000 + , Dunedin, FA, USA) with a 420 nm long-pass filter. The ultrafine PM particles in frozen tissues were imaged using a laser scanning microscope (FV300/IX71, Olympus, Japan) equipped with a femtosecond (fs) pulsed laser (680–1300 nm tunable wavelength, 150 fs, 80 MHz, InSight X3 Dual, USA) and a time correlated single photon counting (TCSPC) system (SPC-150, Becker & Hickl, Germany), with a 60 × water-dipping objective (NA = 1.2). The fluorescence lifetime imaging microscopy (FLIM) were excited by the 830 nm fs laser and collected with a 770 nm (shortpass) SP filter. The ultrafine PM particles in PBS solution were measured by FLIM as well to obtain the fluorescence lifetime of ultrafine PM. Each figure had a field of 123 µm × 123 µm with 256 × 256 pixels, collecting the signal within the depth of about 2 µm. At least 6 different areas were randomly imaged for each sample. The mean lifetime of each pixel is fitted with multi-exponential decay models and calculated using the commercial SPC Image software package (Becker & Hickl GmbH, Berlin, Germany). The fluorescence lifetime of ultrafine PM solution was mostly in the range of 170–200 ps with the peak at 174 ps. Therefore, the FLIM images of tissues with ultrafine PM were fitted by setting the shortest fluorescence lifetime component as 174 ps. Then the pixels with short lifetime were marked as green noting ultrafine PM particles, and the pixels with long lifetime were depicted as red denoting tissue autofluorescence. Ultrafine PM particle density in the lung or heart tissues of dirty air- or FA-treated mice were calculated based on the two-colored images.

### Histological and immunohistochemical staining

The lung and heart tissues were subjected to Congo red staining for histological and morphological information. Congo red dye was obtained from Ruibao and Biotech Co., Ltd (Cat # R1029). For immunohistochemical analyses the following antibodies and materials were used: IBA-1 (Reego and Biology, 1:100), Aβ (Reego and Biology, 1:500). HRP-labelled goat anti-rabbit secondary antibody (Reego and Biology, 1:200), DAB (DAKO, K5007), normal rabbit serum (Boster, AR1010), and BSA (Solarbio, A8020). High resolution optical images of the stained tissues were observed by Olympus CKX53 microscope and recorded by using Olympus cellSens 2.1 [ver.2.1] imaging software for Life Sciences (Olympus, Tokyo, Japan).

### Statistical analysis

All the data were expressed as Mean ± Standard deviation (SD). The difference between PM group and FA group was analyzed using student *t*-test. The statistical analysis was performed using SPSS22.0 software (IBM, Armonk, NY). Graphpad Prism software (Version 6.0, La Jolla, CA) and OriginPro 2021b was used for graph plotting. P < 0.05 was considered significant.

## Supplementary Information

**Additional file 1: Fig. S1**. Fractograms of heavy metals detected in PM particles by ICP-MS. (**A**) Cr, (**B**) Mn, (**C**) Fe, (**D**) Ni, (**E**) Cu, (**F**) Zn, (**G**) Cd, and (**H**) Bi.

**Additional file 2: Table S1**. The mean concentrations of metal elements in dirty air and FA during exposure.

## Data Availability

All data generated or analyzed during this study are included in this published article [and its additional files].
