# Supplementary figures and images for: Label-free detection and quantification of ultrafine particulate matter in lung and heart of mouse and evaluation of tissue injury
Source: Part Fibre Toxicol. 2022 Jul 26;19:51. doi: 10.1186/s12989-022-00493-8 (PMC9316794; doi:10.1186/s12989-022-00493-8)

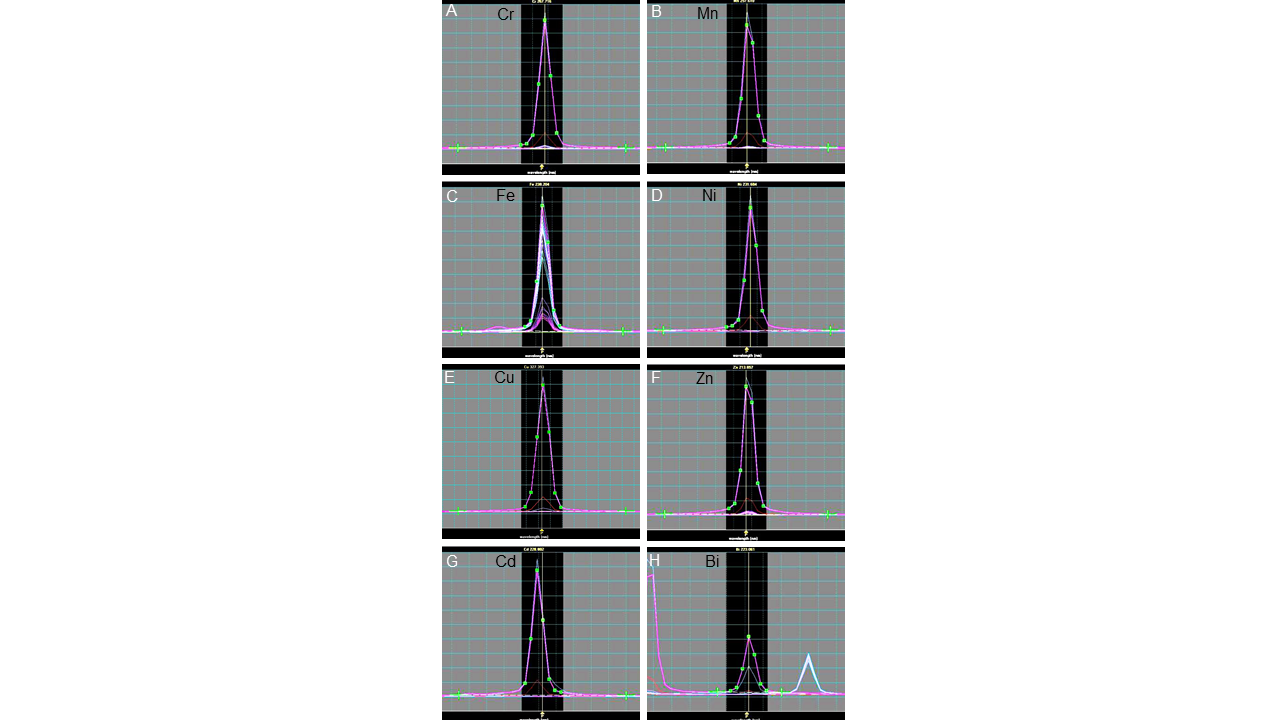

Supplement: Supplementary file 1 — Additional file 1: Fig. S1. Fractograms of heavy metals detected in PM particles by ICP-MS. (A) Cr, (B) Mn, (C) Fe, (D) Ni, (E) Cu, (F) Zn, (G) Cd, and (H) Bi. [file 12989_2022_493_MOESM1_ESM.tif]
