# Supplementary material for: Label-free detection and quantification of ultrafine particulate matter in lung and heart of mouse and evaluation of tissue injury
Source: Part Fibre Toxicol. 2022 Jul 26;19:51. doi: 10.1186/s12989-022-00493-8 (PMC9316794; doi:10.1186/s12989-022-00493-8)
Supplement: Supplementary file 2 — Additional file 2: Table S1. The mean concentrations of metal elements in dirty air and FA during exposure. [file 12989_2022_493_MOESM2_ESM.docx]

**Supplementary Material**

**The metal components of ultrafine PM particles in dirty air and FA chambers**

**Table S1 The mean concentrations of metal elements in dirty air and FA during exposure**

| **Metals** | **Dirty air** **(ng/m^3^)** | **FA (ng/m^3^)** | **Dirty air/FA** |
| --- | --- | --- | --- |
| Zn | 78.34 ± 0.27 | 15.43 ±1.15 | 5.08 ±1.04 |
| Bi | 26.42 ± 0.31 | 5.36 ± 0.81 | 4.93 ± 0.92 |
| Cd | 31.21 ± 0.43 | 8.27 ± 1.28 | 3.77 ± 0.56 |
| Ni | 7.27 ± 1.14 | 2.53±0.26 | 2.87±0.14 |
| Fe | 161.52 ± 7.16 | 32.18 ± 6.39 | 5.02 ± 1.51 |
| Mn | 47.83 ± 4.37 | 11.06 ± 2.54 | 4.32 ± 1.35 |
| Cr | 38.12 ± 3.56 | 8.11 ± 2.83 | 4.70 ± 0.67 |
| Cu | 18.25 ± 1.34 | 4.22 ± 0.85 | 4.32 ± 0.58 |
